# Medulloblastoma and the DNA Damage Response

**DOI:** 10.3389/fonc.2022.903830

**Published:** 2022-06-07

**Authors:** Leon F. McSwain, Kiran K. Parwani, Shubin W. Shahab, Dolores Hambardzumyan, Tobey J. MacDonald, Jennifer M. Spangle, Anna Marie Kenney

**Affiliations:** ^1^ Department of Pediatrics, Emory University, Atlanta, GA, United States; ^2^ Winship Cancer Institute, Emory University, Atlanta, GA, United States; ^3^ Department of Radiation Oncology, Emory University, Atlanta, GA, United States; ^4^ Departments of Neurosurgery and Oncological Sciences, Icahn School of Medicine at Mount Sinai, New York, NY, United States

**Keywords:** medulloblastoma, radiation oncology, pediatrics, therapeutic targeting, p53 status

## Abstract

Medulloblastoma (MB) is the most common malignant brain tumor in children with standard of care consisting of surgery, radiation, and chemotherapy. Recent molecular profiling led to the identification of four molecularly distinct MB subgroups – Wingless (WNT), Sonic Hedgehog (SHH), Group 3, and Group 4. Despite genomic MB characterization and subsequent tumor stratification, clinical treatment paradigms are still largely driven by histology, degree of surgical resection, and presence or absence of metastasis rather than molecular profile. Patients usually undergo resection of their tumor followed by craniospinal radiation (CSI) and a 6 month to one-year multi-agent chemotherapeutic regimen. While there is clearly a need for development of targeted agents specific to the molecular alterations of each patient, targeting proteins responsible for DNA damage repair could have a broader impact regardless of molecular subgrouping. DNA damage response (DDR) protein inhibitors have recently emerged as targeted agents with potent activity as monotherapy or in combination in different cancers. Here we discuss the molecular underpinnings of genomic instability in MB and potential avenues for exploitation through DNA damage response inhibition.

## Current Clinical Approaches to Medulloblastoma

**Figure 1:**
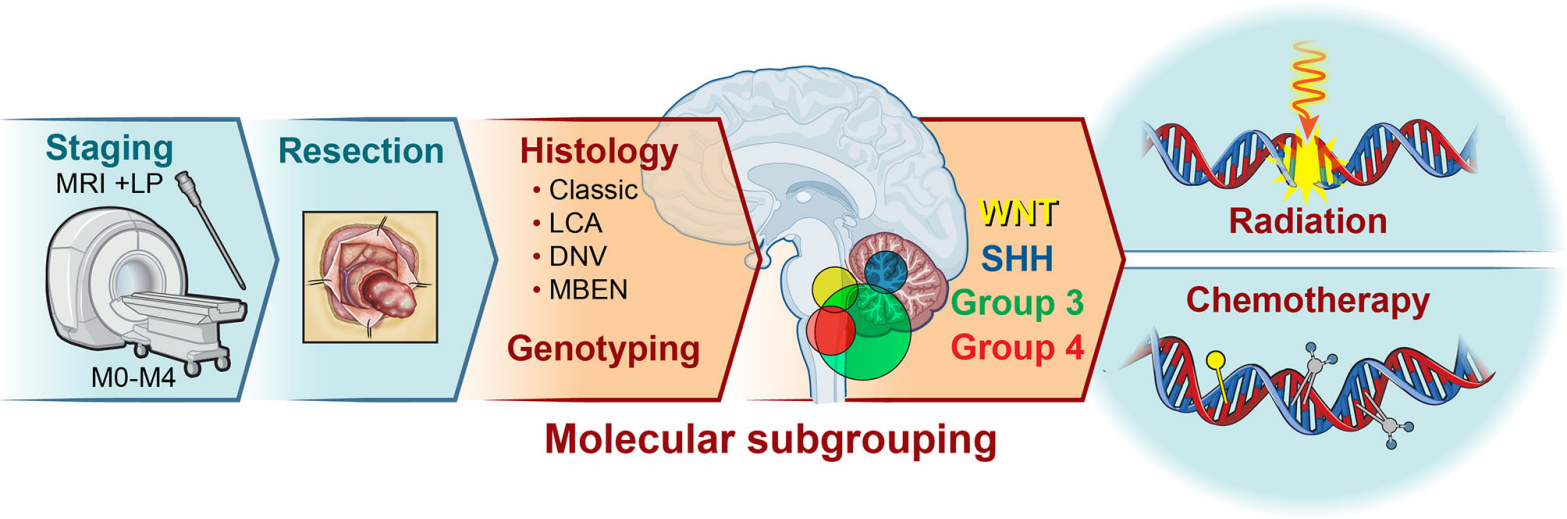
Medulloblastoma is a tumor of the cerebellum. Medulloblastoma constitutes the most common childhood malignant brain tumor, accounting for approximately 20% of all central nervous system tumors. Patient stratification consists of categorization based on age and presence of metastasis, histology, and genotyping. Following initial staging*, maximal surgical resection is typically attempted for all patients. Subsequent histology and molecular profiling introduces subgroup classification and potential alterations to the treatment protocol. While the vast majority of patients receive some combination of radiation and chemotherapy, the age, location of tumor (2), extent of spread, histology, and subgroup inform the intensity and duration of treatment. MRI, Magnetic Resonance Imaging; STR, Subtotal Resection; GTR, Gross Total Resection; LCA, Large Cell Anaplastic; DNV, Desmoplastic/Nodular; MBEN, medulloblastoma with extensive nodularity; LP, Lumbar Puncture *may be delayed to post-surgery.

Medulloblastoma (MB) is the most common malignant brain tumor affecting children. Standard treatment of non-infant medulloblastoma requires maximal safe surgical resection followed by radiation and chemotherapy (1). Within the last ten years, microarray and methylome profiling from multiple groups led to the identification of four major molecularly distinct MB subgroups – Wingless (WNT), Sonic Hedgehog (SHH), Group 3, and Group 4 (**Figure 1**) (3–5). While WNT and SHH MB are driven by alterations in WNT and SHH pathways respectively, oncogenic drivers of Group 3 and Group 4 tumors are less clear (6, 7). The amplification of c*MYC*, *MYCN*, and mutations in *TP53*, are common molecular alterations driving DNA damage induced apoptotic resistance and frequently enriched upon relapse (6, 8). While c*MYC* amplification is predominant in Group 3 MB and is associated with poor patient prognosis, this occurs in other subgroups as well (9). Furthermore, following SHH stratification into 4 molecularly distinct categories (SHHα SHHβ SHHδ SHHγ) it was found that p53 mutations are enriched in SHHα and patients have a worse prognosis (10). Thus, prognosis varies based on subgrouping and in fact WNT patients have the best outcome compared to those of other subgroups with *MYC* amplification or *TP53* mutation (11).

Despite recent genomic MB characterization and subsequent tumor stratification, clinical treatment paradigms are still largely driven by histology, degree of surgical resection, and presence or absence of metastasis rather than molecular profile (12). Patients usually undergo resection of their tumor followed by craniospinal radiation (CSI) and a 6 month to one-year multi-agent chemotherapeutic regimen. Traditionally, radiation has been recognized as the mainstay of treatment for non-infant MB patients (>3 yo). Prior to the 1950s, MB was considered a universally fatal diagnosis. In 1953, Patterson and Farr published a case series of 27 patients treated with CSI and observed a 3-year survival of 65% (13). This landmark paper led to the widespread acceptance of CSI as the therapy for MB. However, the long-term neurocognitive sequelae of radiation therapy, along with the advent of cytotoxic agents, propelled the addition of chemotherapy in the 1970s (14). Since then improvement in survival has been incremental, comprising better surgical and radiation techniques, better supportive care, and combination/intensification of agents.

Over the last decade, modifications to the above therapeutic regimen have been primarily driven by molecular subgrouping. Given the good outcomes of patients with WNT tumors, there is an effort to reduce the radiation dose for these patients (12). Additionally, administration of carboplatin concomitant with radiation was recently found to be beneficial specifically for Group 3 patients (15). Multiple clinical trials are underway to evaluate the efficacy of targeted therapies, particularly in SHH MB, although none have made it to clinical practice yet. While there is clearly a need for development of targeted agents specific to the molecular alterations of each patient, targeting proteins responsible for DNA damage repair could have a broader impact regardless of molecular subgrouping. DDR protein inhibitors have recently emerged as targeted agents with potent activity as monotherapy or in combination in different cancers. Historically, the synthetic lethality achieved through PARP inhibition in *BRCA1/2* mutated breast cancer represents the ideal scenario for DDR inhibitor monotherapy; however, PARP inhibition is applicable outside of *BRCA1/2* mutated cancers (16). In the setting of MB, the use of DDR inhibitors in conjunction with standard of care therapies represents an avenue for sensitization to lower doses of standard of care therapies even without synthetically lethal combinations. In this review we discuss the components of the DDR as they relate to MB and the potential for therapeutic benefit using DDR inhibitors, either alone or in rational combinations.

## DNA Damage Signaling

Since the standardization of medulloblastoma treatment from the mid-1980s to the mid-2000s, surgical resection, radiation and chemotherapy continue to serve as the primary treatment modalities for MB patients (17, 18). While radiation delivery optimization and dose reduction preserves vital tissue proximal to the primary tumor site, posterior fossa irradiation and on-target off-tumor effects of chemotherapy remain important considerations for therapeutic development (19, 20). Targeting proteins involved in resolving DNA damage from either ionizing radiation (IR) induced single and double strand breaks (SSBs and DSBs) or adducts formed by chemotherapies could permit lower therapeutic dosing and more effective targeting of tumor cells (21).

## Targeting Proteins Involved in the DNA Damage Response to Radiation and Chemotherapy

**Figure 2:**
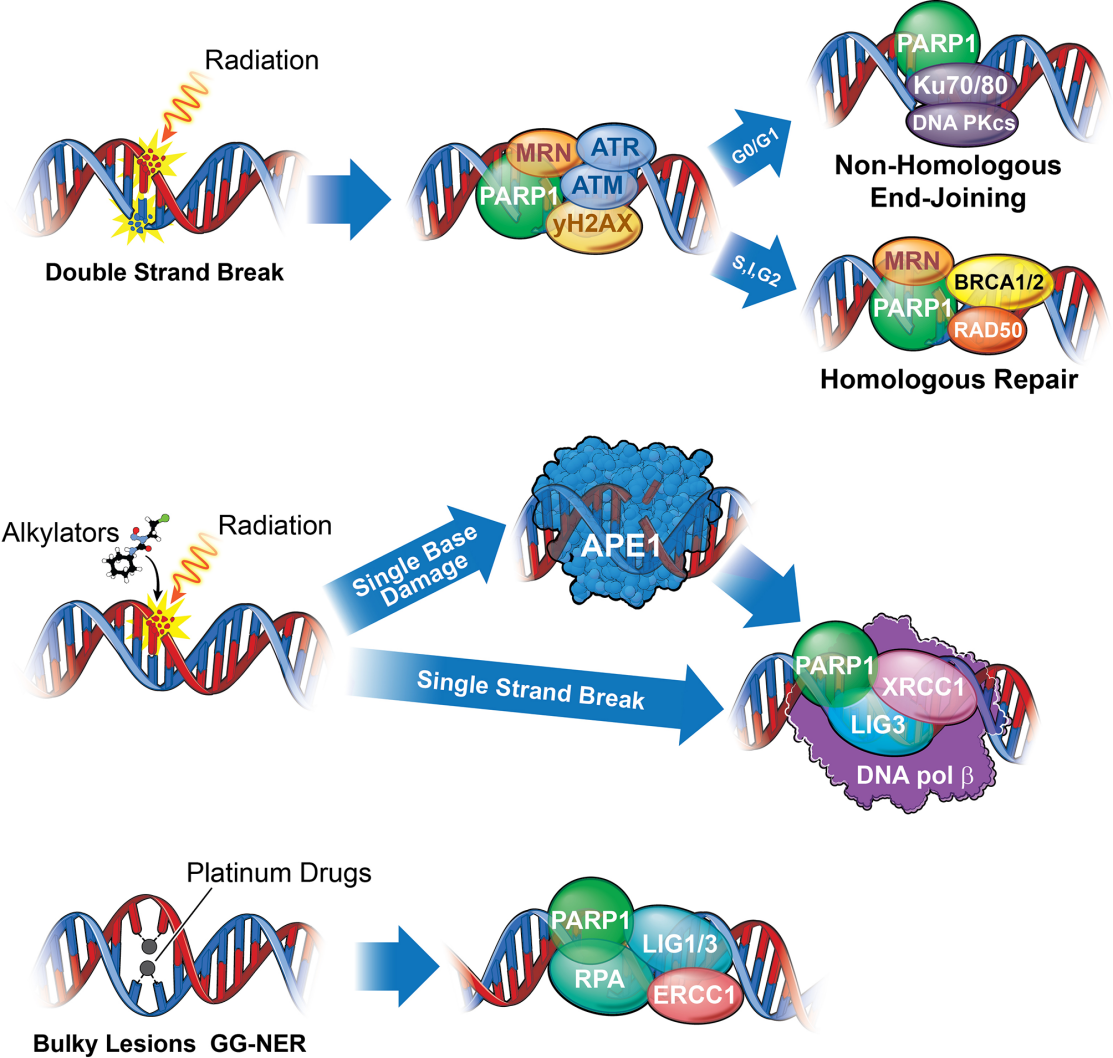
A summary of the DNA damage response in medulloblastoma. Numerous DNA damage response signaling axes mediate DNA repair following medulloblastoma standard of care. Chemotherapies utilized, including temozolomide, cisplatin, lomustine, and cyclophosphamide (with cisplatin and cyclophosphamide constituting standard of care), bind directly to DNA to create bulky lesions repaired through a combination of either Base Excision Repair (BER), Nucleotide Excision Repair (NER), MGMT (Not shown), and HR or NHEJ, while IR mediated DSBs are repaired through NHEJ and HR. (Top) The deleterious effects of Ionizing radiation results primarily from DNA double strand breaks (DSBs) repaired either through Homologous Repair (HR) or Non-Homologous End Joining (NHEJ). While PARP, M/R/N (a complex of 3 proteins: MRE-11, RAD50, NBS), and ATM function universally to recognize strand breaks, cells in G0/G1 lack sister chromatids and will repair through a more error prone NHEJ, while those in S, Interphase, or G2 will repair through HR, utilizing the sister chromatid to replace the missing bases. Proteins directly involved in repair of double strand breaks include Ku70/80 and DNA-PKcs for NHEJ and M/R/N, BRCA1/2, and RAD50 for HR. Upstream effectors, ATM and ATR, will signal through Chk1 and 2 to arrest the cell and either repair or commit apoptosis (**Figure 4**). (Middle) Single strand breaks resulting from radiation or excision of damaged base pairs by APE1 (such as those resulting from alkylation) are identified by PARP1 and repaired by XRCC1, DNA Ligase III (LIG3), and DNA Polymerase β. (Bottom) Bulky lesions resulting from platinum-based drugs such as cisplatin or carboplatin can be repaired either through transcription coupled NER (TC-NER) or global genomic NER (GG-NER). While it remains unclear what role PARP may play in TC-NER, it serves as a recognition and recruitment protein for GG-NER, functioning alongside DNA Ligase I or III (LIG1/3), RPA, and ERCC1 proteins. Bifunctional alkylating agents and chloro-ethylating agents, such as cyclophosphamide or lomustine, respectively, can methylate guanine. Methylated guanine can be repaired through MGMT (not shown), through direct removal of guanine methyl group, potentially resulting intrastrand cross-linking (ICL) requiring a combination of NER and HR or NHEJ to repair) or interstrand cross-linking, repaired through NER. Platinum drugs mediate interstrand cross-linking, repaired predominantly through NER. Finally, temozolomide can be repaired either through BER (requiring APE1) or MGMT (24).

The DNA damage responses to IR-induced SSBs and DSBs, and adducts formed by chemotherapies, are extensively reviewed elsewhere (22, 23). Here we will discuss the central tenets of the DDR, including detection, downstream signaling, and repair, as they pertain to MB therapy resistance and potentially targetable DDR proteins. Firstly, exposure to ionizing radiation results in the accumulation of SSBs and DSBs, which the cell will repair through one of the two methods: homologous recombination (HR) or the more error prone non-homologous end joining (NHEJ) (**Figure 2**) (25). Comparatively, DNA adduct forming agents, such as those commonly used to treat MB including cisplatin, lomustine, cyclophosphamide, and temozolomide, require alternate repair proteins to perform base excision repair (BER) or nucleotide excision repair (NER) (**Figure 2**). Proteins important for detection, signaling, and repair, including ATM/ATR, CHK1/2, and PARP function in both types of damage repair while APE1 and CK2 facilitate DNA adduct repair, specifically. Other proteins discussed include AKT, a mediator of radiation resistance in the MB stem cell niche, p53 the “guardian of the genome,” which when mutated, results in substantially worse standard of care response primarily due to defective cell death signaling downstream of damage recognition, and proteins that regulate p53.

## ATR-Chk1

Dozens of proteins function together to recognize and initiate repair of damaged DNA; however, ATR and ATM can initiate cell cycle arrest, damage repair, and apoptosis by signaling through Checkpoint Kinase 1 and 2 (**Figures 1**, **4**) (26). More specifically, the ATR-Chk1 signaling axis has emerged as a key player in postnatal cerebellar development and MB therapeutic resistance, and Chk1 is upregulated across MB subgroups (27). Saran and colleagues investigated SHH signaling in a patched 1 receptor heterozygous IR inducible SHH MB model (hereafter referred to as the *PTCH^+/-^* model), where radiation induces a “second hit” to the smoothened receptor inhibitor, *PTCH*, resulting in constitutive SHH signaling and tumorigenic transformation. The group uncovered a link between *PTCH* heterozygosity and tumor formation following IR exposure in postnatal day 4 (P4) but not P10 mice as a result of differences in p53 activation (28, 29). Subsequently, SHH pathway dysregulation was linked to ATR-Chk1 pathway inactivation in the *PTCH^+/-^* model (30). Through overactivation of the SHH signaling cascade *via PTCH* inactivation and overexpression of Gli1 protein, the phosphorylation and activation of Chk1 is attenuated, resulting in abrogation of the S-phase cell cycle checkpoint and chromosomal aberrations. In an ATR-deleted mouse model (*ATR^hGFAP-Cre^*) of cerebellar granular neural precursor cells (CGNPs), the putative cell of origin for SHH MB, cells develop extensive chromosomal abnormalities leading to cerebellar hypoplasia (31). From the same study, ATR deletion results in abrogation of cell cycle checkpoint activation, evidenced by PCNA and pHH3 positive staining, and accumulation of γH2AX, a marker of DNA damage, ultimately resulting in p53 accumulation, caspas-3 cleavage, and apoptosis. Additionally, ATR deletion from a smoothened overexpressing SHH model through cre-recombination (*SmoM2;Atr^G-cre^*), inhibits tumor formation; all of which suggest a requirement for ATR in maintaining genomic stability during cerebellar development and tumor formation, nominating the ATR-Chk1 axis as a potential therapeutic target.

What we ultimately seek to therapeutically exploit by targeting the ATR-Chk1 pathway are cells with unstable genomes; in fact, many cells amenable to ATR-Chk1 signaling inhibition are *MYCN* or c*MYC* amplified or overexpressing. SHH and WNT subgroups overexpress *MYCN* while Group3 and WNT subgroups overexpress *cMYC* (9). *MYCN* and *cMYC* increase the number of firing replication origins, causing collision between replication and transcriptional complexes, resulting in fork stalling and collapse and the accumulation of DDR marks at the sites of active DNA replication (32–34). However, the simultaneous regulation of cell cycle check-points and DDR allows cells to maintain a complex balancing act between genomic instability and tumor maintenance, which depends upon the ATR-Chk1 signaling axis (34). An example of this can be found in *MYCN* expressing SHH MB. Sonic Hedgehog signaling drives *MYCN* expression in both CGNPs and MBs of the *PTCH^+/-^* model, and *MYCN* overexpression can drive cell proliferation independent of SHH signaling (35). Coincidently, the genomic instability resulting from *PTCH1* deletion can lead to the amplification of regions of chromosome 12, including the *MYCN* gene (36). Through transcriptional upregulation of the MRE11/RAD50/NBS1 (M/R/N) complex downstream of ATR-Chk1 in CGNPs, *MYCN* drives not only a genomically unstable, highly replicative state, but also an increase in proteins required for recognition and repair to counter the instability (32).

The lack of enzymatic activity and hydrophobic pockets paired with an intrinsically-disordered N-terminal make *MYCN* a challenging drug target (37). Given these challenges, alongside the potentially broader impact of DNA repair protein inhibition, much of the research has focused on sensitization of cells to genomic stress through inhibition of either Chk1 or Wee1, a target of Chk1 (38). The correlative relationship between protein expression and cell sensitivity to protein inhibition is not applicable in the case of Chk1. While all MB subgroups demonstrate elevated Chk1 expression and worse prognosis with high Chk1 expression, as mentioned previously, the relative expression of *cMYC* is a greater predictor of responsiveness to Chk1 inhibition (27). In earlier studies utilizing AZD-7762, a Chk1 inhibitor, numerous cell lines demonstrated response as measured through reductions in cell viability, accumulation of γH2AX, and increase in apoptotic markers. Daoy, D283, D425, UW-228, and HD-MB03 all show varying degrees of Chk1 inhibitor response; however, high *cMYC*-expressing cells such as D283 and HD-MB03, both Group 3 cell lines, are more sensitive to damage as seen through a higher ratio of reduced viability to γH2AX foci formation (27, 39). Lower levels of damage accumulation are required to push the cell fate towards apoptosis. *In vivo* and synergism studies subsequently emerged using AZD-7762, MK-8776, Rabusertib, or Prexasertib (all Chk1 inhibitors) in combination with cisplatin or gemcitabine. Inhibition of Chk1 in Group 3 cells, including D425, D283, and SU-MB002, but not SHH PDX models, results in cell cycle checkpoint abrogation and a greater accumulation of DNA damage and subsequent cell death following simultaneous exposure to cisplatin or gemcitabine (40).

Genomic surveillance is crucial for cerebellar development, tumorigenesis, and tumor maintenance. While ablation of the ATR-Chk1 axis results in cerebellar hypoplasia in non-tumor models, downregulation during SHH tumor development leads to extensive chromosomal abnormalities and abrogation of tumor development. This complex balancing act is a requirement for maintaining a highly proliferative, tumorigenic state. The *cMYC* amplification and overexpression of Group 3 models creates a reliance on the ATR-Chk1 axis for viability where inhibition could result in cell death, a phenotype potentially exploitable in WNT tumors given their MYC status. And even though SHH PDX models do not appear responsive to CHK1 inhibition in recent studies, inhibition of MYCN → M/R/N signaling is unexplored. *MYC* amplification alongside Chk1 upregulation may serve as reliable biomarkers for Chk1 pathway inhibition, particularly in Group 3 patients who have the worst prognosis. Currently, Prexasertib in combination with Cyclophosphamide or gemcitabine is in a phase 1 clinical trial for refractory Group 3 and Group 4 patients.^1ct^

## PARP

Poly-ADP-ribose Polymerase, or PARP, plays numerous roles in the DNA damage response, but is most notable for damage recognition and repair protein recruitment through ribosylation of damaged DNA and auto-ribosylation. While PARP1 is not typically required for cell survival, because it facilitates repair of IR induced strand breaks through both NHEJ and HR, and repair of bulky adducts through NER (**Figure 2**), inhibition could sensitize MB cells to existing therapies and even synergize with the aforementioned Chk1 inhibitors (41–43). In fact, while *BRCA1* and *BRCA2* mutation status are positive predictors of PARP1 inhibitor response due to synthetic lethality in the setting of other tumor types, this is not a requirement and other gene signatures can serve as predictors of therapeutic response where the use of single molecular markers prove to be insufficient (44). Additionally, the *BRCA1/2* mutation status across MB patients remains unclear, though RNAseq data have shown upregulation of gene signatures associated with *BRCA1/2* mutation in Group3 and 4 and a subset of SHH patients (45).

A role for PARP1 in MB tumor formation emerged through studies of p53 null mice. While *TP53* ablation alone is not sufficient for brain tumor formation, in *TP53* ^-/-^; *PARP1* ^-/-^ mice, neuronal cells are predisposed to malignant transformation (46, 47). Saran and colleagues, using a p53 wild type *PTCH^+/-^;PARP1^-/-^* model of SHH MB, demonstrated that *PARP1* ablation leads to increased frequency of preneoplastic lesions, accumulation of γH2AX foci, and CGNP genomic instability (48). Chromosomal rearrangements resulting from *PARP1* abrogation lead to a second hit in the *PTCH1* allele, increasing the incidence of tumor formation. The absence of PARP also increases phosphorylation of Ser18-p53, suggesting that the majority of genetically unstable cells undergo apoptosis while few cells escape to accelerate tumor formation. Pre-treatment of *in vitro* MB models, D283, D556, and UW228-2, with olaparib prior to irradiation results in greater accumulation of γH2AX foci that are sustained longer than control (49). In fact, olaparib is blood brain barrier (BBB) penetrant and could serve as an effective brain tumor therapy (50) Considering the genomic instability of CGNPs following *PARP1* and *ATR* ablation, there is also a potential for synergism between PARP and ATR inhibition. While not yet studied in the setting of MB, in glioma-bearing mice the combination of VE822, an ATR inhibitor, and olaparib leads to a 60% increase in survival compared to control-treated (51).

In addition to PARP’s role in radiation induced strand break repair, it also mediates base excision repair. As mentioned above, PARP1 is critical for resolution of adducts by functioning as a component of the BER complex, consisting of DNA ligase III (*LIG3*), DNA Polymerase β *(POLB)*, and XRCC1 (41, 52). Adducts formed by lomustine, cisplatin, and cyclophosphamide, which serve as cytotoxic chemotherapies to treat MB, require functional BER or NER for resolution (53, 54). Unfortunately, the combination of PARP inhibitors with cyclophosphamide does not improve response rate over cyclophosphamide alone, at least in the setting of breast cancer, and combinations with lomustine are understudied (55). However, the administration of PARP inhibitors alongside platinum-based drugs like cisplatin has the potential to enhance targeting of tumor cells while reducing secondary cytotoxicities (56). In fact, compared to veliparib-mediated catalytic inhibition of PARP1, which abrogates PARylation, olaparib demonstrates superior DNA-PARP trapping, resulting in not only a lack of repair, but also replication fork stalling and double strand breaks. Olaparib synergizes with cisplatin and temozolomide, an emerging MB therapeutic for recurrent patients (57–60). In Group 3 and 4 xenograft models generated using D384, D425, and D283 cell lines, D384 and D425 show a robust response to combination therapy with temozolomide and another PARP inhibitor rucaparib, whose DNA trapping kinetics are comparable to those of olaparib (61, 62).

Historically, PARP inhibition induced apoptosis through synthetic lethality when combined with *BRCA1/2* mutations in breast cancer patients resulting from unrepaired strand breaks. However, *BRCA1/2* mutation is not a requirement for the use of PARP inhibitors. PARP1 is crucial to DDR activation through its PAR catalytic activity, which is exemplified in developmental MB models where ablation leads to genomic instability and cell death following radiation. While there is a reliance of MYC amplified cells on the ATR-Chk1 signaling axis, PARP inhibition appears to be relevant across all subgroups not only in the context of radiation but also when combined with platinum-based drugs and temozolomide. Inhibiting PARP could have a broader impact compared to ATR-CHK1 in cells without *MYC* amplification or overexpression in combination with standard of care therapies and could afford dose decreasing to ameliorate toxicities from chemotherapy, namely cisplatin. Currently, olaparib is in a phase 2 clinical trial for patients with advanced or refractory solid tumors, including MB.^2ct^

## APE1

Apurinic/apyrimidinic endonuclease 1 (APE1) not only regulates DNA binding of transcription factors through cysteine residue redox regulation, it is also responsible for DNA incision proximal to adducts formed by platinum-based drugs and alkylating agents such as cisplatin and temozolomide (**Figure 2**) (63, 64). The production of oxygen free radicals from ionizing radiation also produces abasic sites in the DNA reparable through APE1, the abrogation of which leads to unrepaired DNA and cell death (65–67). Additionally, due to the potential for PARP inhibitor resistance, targeting APE1 could sensitize PARP inhibitor resistant cells to chemotherapy and radiation (68).

Similar to a requirement for PARP and ATR in maintaining genome integrity during cerebellar development, due to the high CNS oxidative stress in postnatal mice, APE1 can protect cells against postnatal oxidative DNA damage (69). 100% of Mice lacking APE1 die within 30 days of birth concomitant with accumulation of extensive γH2AX accumulation. And while p53 ablation in these mice rescues their viability, the absence of p53 alone does not result in tumor burden by post-natal day 20 whereas 100% of (Ape1^L/L^;p53^L/L^)^Nes-cre^ mice develop tumors by day 15. From the same study, APE1 deficient astrocytes maintain radiation induced DNA damage compared to controls and are sensitized to cisplatin. Similarly, after uncovering a role for APE1 in mediating resistance of glioma to adjuvant radiation and alkylating agent based chemotherapy, Silber and colleagues extended these findings to MB and primitive neuroectodermal tumors (70–72). In patients deemed high risk as indicated by tumor invasion into the surrounding brain, APE1 endonuclease activity is elevated (73). In UW228-2 cells treated with siRNA against APE1 in combination with temozolomide, survival is decreased compared to control. These data point to a requirement for APE1 to mediate cisplatin adduct resolution in MB. Given the barriers to small molecule inhibitor development for some targets and potential lack of BBB penetrance, the utilization of siRNA conjugated nanoparticles has emerged as a viable alternative, potentially alleviating inhibitor resistance and issues with BBB penetrance (74). UW228 cells exposed to nanoparticle conjugated siRNA against APE1 *in vitro* sustain radiation induced DNA damage (69, 75). Only recently did a selective APE1 inhibitor emerge in high throughput drug screening of non-small cell lung cancer (NSCLC) (76). The application of NO.0449-0145 induces DNA damage *in vitro* and in an *in vivo* xenograft model and overcomes cisplatin and erlotinib resistance; however, the BBB penetrance and applicability in MB would require further investigations.

## CK2 and MGMT

Casein Kinase II (CK2), a pleiotropic protein with diverse functions from Epithelial to Mesenchymal Transition (EMT) regulation to DNA damage repair, has become a potential therapeutic target in MB (77). While CK2 regulates redox activity of APE1 through post-translational modification, its role in DNA repair comes from signaling through O6-MethylGuanine-DNA Methyltransferase (MGMT), a DDR protein capable of mediating temozolomide resistance (78–80). Unlike protein complexes that mediate BER and NER, MGMT can function alone to repair *O*6-AG and *O*4-alkylthymine adducts formed by alkylating agents (81). Even though MGMT promoter methylation in CpG rich sequences and subsequent overexpression is well known for mediating temozolomide resistance in glioblastoma, the clinical relevance of targeting MGMT in MB has only recently emerged (82).

In 2018 Li and colleagues uncovered a CK2 → β-Catenin → MGMT signaling axis in SHH MB (80). Knockout of either isoform, *CSNK2A1* or *CSNK2B*, results in decreased tumorigenic potential. In a high throughput screen using CX-4945, an orally bioavailable and BBB penetrant selective inhibitor of CK2, temozolomide was found to synergize with CK2 inhibition (83, 84). Indeed, CK2 KO leads to a decrease in MGMT and β-Catenin and an increase in apoptosis following temozolomide exposure, which was rescued by re-expression of β-Catenin. These data point to CK2 as a modulator of MGMT activity and temozolomide resistance in MB. There are other DDR proteins involved in recognition, repair, and signaling found to be CK2 substrates, including p53, BRCA1, XPB, XRCC1, XRCC4, Histone H1, and Rad51 (85). CK2 is also implicated in driving proliferative and migratory phenotypes and inhibition of apoptosis in multiple cancers, including MB (85). Initial studies showed binding of CK2 to the smoothened receptor and promotion of SHH signaling (86). Proteomic analysis of CGNPs showed increased phosphorylation of CK2 motifs in postnatal day 7 mice, implicating CK2 as a driver of cerebellar developmental programming (87). Subsequent CK2 knockdown or treatment with the CK2 inhibitor 4,5,6,7-Tetrabromobenzotriazole (TBB) destabilizes Gli2 and decreases Gli1 expression, downstream mediators of SHH signaling. More importantly, D175N mutation in CK2 confers resistance to TBB but not CX-4945 likely due to ATP-binding cavity enlargement, further demonstrating CX-4945 robustness. These findings point to a broader impact of CK2 targeting and the potential for radiation and chemotherapy sensitization and inhibition of oncogenic phenotypes. CX4945 is currently in a multi-phase clinical trial for recurrent, SHH subgroup MB.^3ct^

## AKT and PI3K

**Figure 3:**
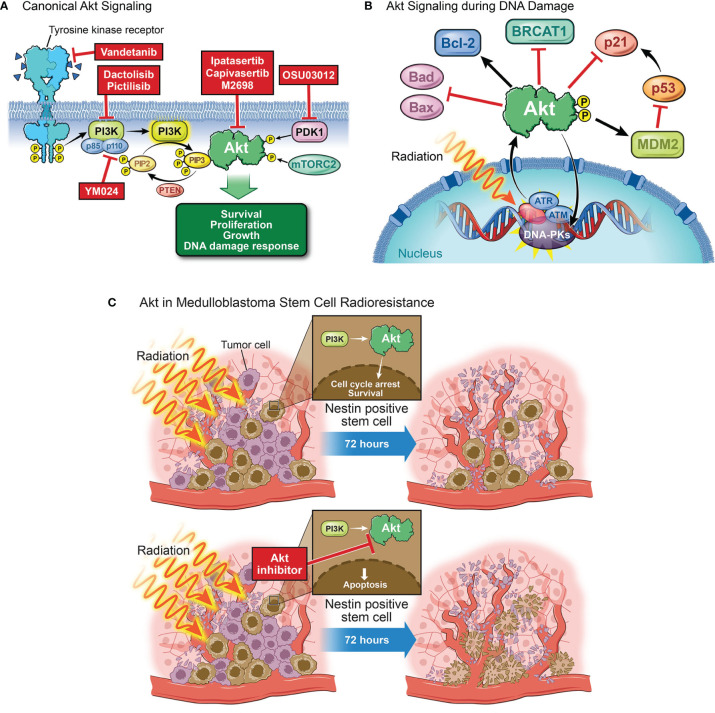
The role of AKT in DNA damage. **(A)** Canonical AKT signaling begins with the activation of a receptor tyrosine kinase (e.g., VEGFR, EGFR, or IGFR). Receptor activation, by binding of a ligand, dimerization, and autophosphorylation, causes binding and activation of p85 and p110, the respective regulatory and catalytic subunits of PI3K. Activated PI3K will go on to catalyze the conversion of PIP2 to PIP3, a process which can be reversed by the tumor suppressor PTEN. If PIP3 is formed, it will recruit AKT to the membrane at which point AKT will be phosphorylated by PDK1 and mTORC2. This dual phosphorylation fully activates AKT, allowing it to affect several downstream processes such as cell survival, proliferation, growth, and the DNA damage response. **(B)** Following exposure to radiation, repair proteins (e.g. ATM, ATR, DNA-PKs) are recruited to the damaged site to facilitate repair. The activation and recruitment of these proteins correlates with an increase in phosphorylated AKT. p-AKT then inhibits apoptosis through Bad and Bax inhibition and Bcl-2 activation. p-AKT will also inhibit the downstream target of p53, p21, to inhibit cell cycle arrest. A feedback loop exists in that AKT can also phosphorylate and activate these proteins to aid in DNA repair. AKT facilitates NHEJ by phosphorylating DNA-PKs and inhibits HR by promoting cytoplasmic localization of BRCA1 and Rad51. **(C)** Irradiation decreases the population of tumor cells at the perivascular niche with the exception of stem cells which are largely radioresistant. Nestin-positive stem cells in the perivascular niche treated with radiation activate the PI3K/AKT pathway to undergo cell cycle arrest and re-enter the cycle 72 hours later, driving MB survival and recurrence. AKT inhibition prior to irradiation abrogates perivascular stem cell radiation resistance, leading to apoptosis of these cells.

AKT, or protein kinase B (PKB), is a member of the AGC serine/threonine kinase family that plays major roles in cellular growth, survival, and DNA repair (88, 89). Receptor tyrosine kinases (RTKs) such as PDGFR, VEGFR, and IGF-1R are some of the primary drivers of AKT phosphorylation in tumors, including MB, typically due to the amplification of the receptors or ligands, or the presence of an autocrine feedback loops (90–94). Overexpression or following ligand binding to these receptors leads to dimerization, autophosphorylation, and activation of phosphoinositide 3-kinase (PI3K) which is recruited to the receptor and activated (95). PI3K then catalyzes the conversion of PIP2 to PIP3, recruiting AKT *via* its pleckstrin homology domain to be phosphorylated by PDK1, on Thr308, and mTORC2, on Ser473 (**Figure 3**) (96, 97). Dual phosphorylation at these marks is a requirement for full activation of AKT, leading to downstream activation of mTORC1 and modulation of cell growth and proliferation (98).

In the context of DNA damage, ATM, ATR, and DNA-PKs can all phosphorylate and activate AKT even in the absence of upstream RTK activation; and while the mechanism through which ATM and ATR activate AKT is unclear, DNA-PKs can directly phosphorylate AKT at serine 473 (99). Additionally, PARP indirectly releases AKT from SIRT1 inhibition and accumulates adenosine monophosphate (AMP) driving AMP-activated protein kinase (AMPK) mediated activation of AKT. AKT can also modulate DDR through feedback mechanisms involving DNA damage signaling sensors and effectors (99). Activated AKT increases *cMYC* transcription and inhibits the cyclin-dependent kinase inhibitor p21^Cip1^, a target of p53, both of which promote cell cycle progression (100, 101). Additionally, AKT suppresses ATR/Chk1 signaling and subsequent HR, yet AKT can be activated in an ATM/ATR-dependent manner and repair DSBs through DNA-PKcs mediated NHEJ. As many DNA damaging agents target dividing cells (102), it is important to highlight that potential resistance to these agents can occur often due to AKT driven NHEJ (103). p-AKT accumulates in irradiated lung carcinoma and prostate cancer cells and activates DNA-PKs to induce NHEJ (104). Conversely, AKT inhibits HR by mediating BRCA1 and RAD51 cytoplasmic retention (105). Active AKT phosphorylates Bad, Bax, and Bcl-2, inhibiting apoptosis and promoting cell survival (106). Together, these signaling roles drive a pro-repair, pro-survival phenotype that could be exploited therapeutically.

Many of these AKT signaling phenotypes are conserved in the setting of medulloblastoma, including both pro-survival and growth and the DNA damage response. In the CGNP developmental model of SHH MB, SHH signaling drives expression of insulin growth factor (IGF), resulting in an autocrine feedback loop between IGF and IGF1R to promote CGNP proliferation, supporting a role for AKT as a developmental protein (107). Additionally, irradiated P1 and P10 mice harbor increased p-AKT in non-irradiated and irradiated cerebella at P1 compared to P10 (108). There is also interplay between p53 levels and p-AKT activity in that P10 cerebella are more resistant to MB formation and sensitive to radiation-induced cell death due to an increase in p53 expression and lower p-AKT activity. Furthermore, AKT constitutes a source of resistance to smoothened inhibitors and is strongly associated with poor outcomes (109). Indeed, smoothened receptor inhibition alongside PI3K through Vismodegib and Dactolisib, respectively, synergizes to decrease cell viability in both SHH and Group 3 cell models (110). From the same study, combination of cisplatin and Dactolisib, but not Vismodegib, results in significant decrease in viability compared to single agent supporting a role for the PI3K/AKT signaling axis in post-DNA damage survival. Inhibition of the upstream AKT kinase, PKD1, with OSU03012 results in a decrease in p-AKT S473 and a sensitization to both doxorubicin and cyclophosphamide but not temozolomide in Group 3 models (111). Subsequently, it was found that many MB cells and patient samples overexpress the oncogenic PI3K catalytic subunit p110α isoform responsible for phosphatidylinositol phosphorylation. YM024-mediated inhibition of the p110α subunit abrogates doxorubicin-induced AKT phosphorylation at S473, sensitizing cells to doxorubicin (112). Inhibition of AKT phosphorylation by targeting upstream RTKs can also sensitize MB to DNA damage. Treatment of SHH-*TP53*-mutated MB and MYC-amplified MB cells with Vandetanib, a multi-kinase inhibitor targeting RET, VEGFR2, and EGFR, reduces cell migration and cell viability (113). When combined with Pictilisib, a PI3K-inhibitor, AKT phosphorylation and protein levels are decreased compared to monotherapy. Following combination, cells are sensitized to etoposide, a chemotherapy which binds and forms a ternary complex between topoisomerase II and DNA. Thus, targeting AKT directly or indirectly would not only decrease tumor growth but will also sensitize cells to DNA damage.

There is also a role for AKT in mediating post-radiation survival of cancer stem cells residing in the perivascular niche (PVN), the putative cells of recurrence for many brain tumors (114). Following radiation, AKT is phosphorylated and activated in nestin-positive mouse MB stem cells (115). The PI3K/AKT/mTOR axis also regulates hypoxia-inducible factor 1-alpha (HIF-1α) mediated expression of VEGF, leading to the vascularization of the tumor, supporting a role for AKT in the development and maintenance of the PVN stem cell niche (116). These findings are further supported by Hambardzumyan et al. who demonstrated that radiation-treated PVN resident nestin-positive stem cells activate the PI3K/AKT pathway, undergo cell cycle arrest, and re-enter cell cycle 72 hours later (117). Furthermore, inhibiting AKT prior to irradiation sensitizes cells in this niche to treatment, demonstrating that these cells’ inherent radiation resistance can be partially overcome through AKT inhibition.

AKT signaling is vital in cell cycling and proliferation which drive tumorigenesis. These processes render cells resistant to DNA damaging agents by the additional upregulation of DNA damage repair proteins which can result in a feedback loop that activates AKT. Previous studies have clearly demonstrated that inhibiting AKT sensitizes MB to DNA damage *in vitro*, making AKT a compelling target *in vivo* that has the potential to be combined with irradiation and chemotherapy. The challenge in AKT targeting lies in ensuring that inhibitors are potent enough to overcome the activation of upstream proteins, such as the receptor tyrosine kinases that activate AKT. Currently, there are several AKT inhibitors in clinical trials as both monotherapy and combination therapy for other solid tumors. Ipatasertib and Capivasertib treatments result in an increase in progression free survival in patients harboring solid tumors such as breast and ovarian cancers (118). However, despite these treatment options, minor toxicities such as hyperglycemia and skin rash still occurred with nausea and fatigue presented when AKT inhibition was combined with chemotherapy (119). M2698 is a BBB penetrant, ATP-competitive inhibitor of AKT1/3 and p70S6K, a downstream target of AKT, that exhibits significant growth arrest in many solid tumor cell lines. M2698 treated mice orthotopically-implanted with GBM cells exhibit increased survival and reduced brain tumor burden (120). Samotolisib, a PI3K isoform inhibitor, is currently in phase 2 clinical trials for patients with relapsed or refractory advanced solid tumors, including medulloblastoma.^4ct^

## p53

**Figure 4:**
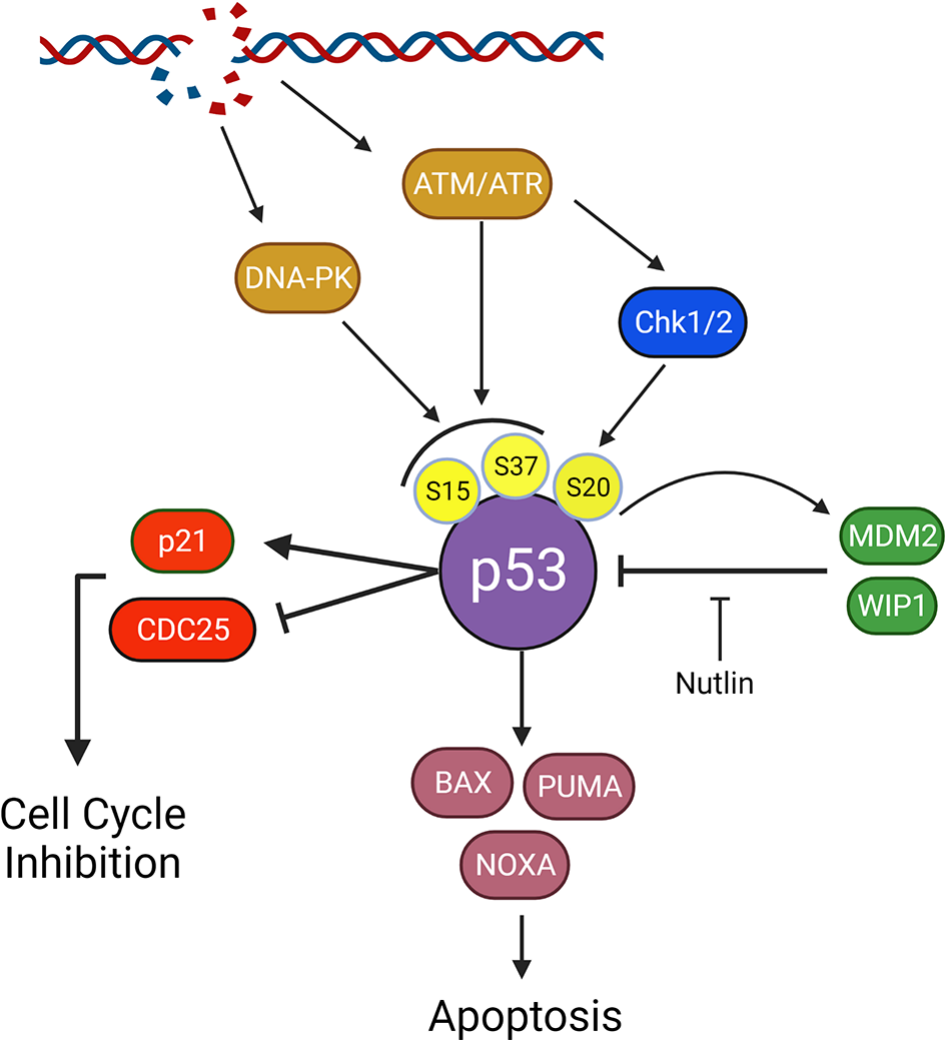
Impact of p53 on survival and p53 signaling in the DNA damage response. P53 is a downstream effector following double strand DNA breaks and plays a crucial role in tumorigenesis. Following Damage detection by ATM/ATR or activation of DNA-PKcs, p53 is phosphorylated at Ser15 or Ser37. ATM and ATR will also activate Chk1 and 2 resulting in phosphorylation of p53 at Ser20. Together these phospho-sites inhibit p53 degradation by preventing MDM2 binding and facilitate p53 tetramerization, a crucial step in p53 activation. p53 will activate p21 and inhibit CDC25 A,B, and C to activate the cell cycle checkpoint and inhibit cycling. p53 will also transcriptionally upregulate pro-death proteins, BAX, PUMA, and NOXA. Upon overcoming inhibition by BCL-2 family apoptotic inhibitors (not shown), BAX, PUMA, and NOXA will activate apoptosis. Inhibition of the MDM2-p53 interaction with nutlin allows for p53 accumulation and apoptosis, ideally in cells heavily reliant on p53 suppression for survival.

p53 expression and mutation status emerged in the early 1990s as a biomarker for poor therapeutic response and shorter overall survival in MB due to the role of p53 in DNA repair and cell fate following genomic insult (**Table 1**) (123–125). In the absence of external cell stressors, p53 is degraded to prevent cell cycle inhibition or inappropriate apoptotic induction; thus, any dysregulation of the p53 pathway resulting from mutations in *TP53* or alterations to proteins responsible for p53 degradation, namely WIP1 and MDM2, is made apparent by atypical alterations to p53 levels (126–128). While *TP53* mutations are infrequent in MB, occurring in approximately 10%-15% of patients, p53 status and the potential for p53 therapeutics even outside of *TP53* mutant patients remain important considerations (8, 129–131). When activated downstream of ATM-Chk2 and ATR-Chk1 following DNA damage, p53 signals through BAX, NOVA, and PUMA to initiate cell death and p21 to inhibit cell cycling, bringing about a potential therapeutic window and an explanation as to why *TP53* mutant patients have a substantially worse prognosis (**Figure 4**) (132).

**Table 1:** p53 mutation status and effect on medulloblastoma patient survival.

Subgroup	Wt p53 Percent	Mut p53 Percent	Wt p535-Year OS	Mut p535-Year OS
SHH	211 Patients^*^ 83.0%	43 Patients^*^ 16.9%	76% ± 4%^*^	41% ± 17%^*^
WNT	83 Patients^*^ 82.2%	18 Patients^*^ 17.8%	94% ± 5%^*^	86% ± 13%^*^
Group 3	72 Patients^*^ 100%	0 Patients^*^ 0%	Avg 54.6%^**^	
Group 4	121 Patients^*^ 99%	1 Patients^*^ 1%	Avg 75.0%^**^	

*161 Zhukova N, Ramaswamy V, Remke M, et al. Subgroup-specific prognostic implications of TP53 mutation in medulloblastoma. J Clin Oncol. 2013;31(23):2927-2935 (121).

**162 Ray S, Chaturvedi NK, Bhakat KK, Rizzino A, Mahapatra S. Subgroup-Specific Diagnostic, Prognostic, and Predictive Markers Influencing Pediatric Medulloblastoma Treatment. Diagnostics (Basel). 2021;12(1). doi:10.3390/diagnostics12010061 (122).

p53 mutant SHH patients respond poorly compared to p53 WT SHH while p53 mutant WNT patients do not show a drastically worse response compared to p53 WT WNT patients. Group3 and 4 patients rarely present with p53 mutations (121). p53 mutations and MYC amplification are present at higher frequencies at relapse and impact patient outcome for all subgroups (not shown) (8).

## *TP53* Status as a Predictor of Chk1 and PARP Inhibition

Given the emergence of DDR specific therapeutics and the role of p53 in mediating cell death and cell cycle checkpoint activation, *TP53* mutation status should be considered as a potential predictor for patient response to inhibition of the previously discussed DDR proteins. While *TP53* wild type CGNPs and mutant Daoy cells demonstrate Chk1 signaling axis requirements, Daoy cells may no longer represent MB patients and targeting Chk1 in novel SHH PDX models shows no survival advantage, regardless of p53 status (27, 32, 39, 40, 133). However, Group 3 PDX models, both *TP53* wildtype, D283, TKMB870, and SU-MB002, and *TP53* mutant cells, D425, in addition to a MYC amplified p53 dominant negative syngeneic mouse model, respond to Chk1 inhibition (40). These data suggest that p53 status is not a predictor of Chk1 response in the setting of Group 3 MB, which is supported by findings in other cancers (134). However, a requirement for p53 functionality in PARP inhibitor mediated cell death in MB is less clear. p53 mediated apoptosis in *PTCH^+/-^* model following PARP ablation suggests that p53 is required for apoptotic induction in the absence of PARP (48). Additionally, the Group 3 models tested for combination of olaparib and radiation, D283 and D556, are *TP53* wild type; and while UW228 is *TP53* mutant and responsive to olaparib and radiation, it may no longer be representative of SHH MB (49, 133). However, when testing combinations of PARP inhibitor and temozolomide in D425 and D384, both Group 3 models, p53 status does not predict PARP inhibitor response, and in fact D425, a *TP53* mutant model, tumor growth inhibition is greater compared to D384 and D283 from the same study (61). Thus, p53 may be required for therapeutic response following PARPi combined with radiation whereas for PARPi plus temozolomide, p53 may not be required.

## p53 and Medulloblastoma Development

p53 plays a significant role in mediating cellular response to radiation and chemotherapy, including induction of apoptosis through BCL-2 and BCL-XL inhibition and transcriptional activation of BAX, NOXA, and PUMA and regulation of G1 and G2 cell cycle checkpoints through numerous targets including p21 and 14-3-3-σ (135, 136). The dysregulation of p53 signaling pathway components introduces mechanisms of therapeutic resistance to standard of care therapy, and, potentially, any of the aforementioned DDR therapeutics. The importance of p53 for apoptosis of pre-neoplastic cerebellum is made apparent through developmental SHH MB modeling where inactivation of p53 alongside other tumor suppressors predisposes to MB development. The absence of p53 and the G1 restriction point protein Rb in CGNPs of the external granular layer leads to induction of MB *via* unabated cell cycling (47). The genomic instability resulting from ablation of these tumor suppressors drives MB development through the amplification of *MYCN* and *PTCH2*, an isoform of the frequently mutated *PTCH1* of SHH MB possessing smoothened inhibitory features (137, 138). Furthermore, in the absence of *PTCH1*, Rb inhibition mediated through ablation of *CDKN2C* (Ink4c or p18), an inhibitor of proteins responsible for Rb inhibition (CDK4, CDK6, and CyclinD1), leads to the formation of MB even in the presence of wild type *TP53* (139). These data provide a mechanism by which loss of p53 or restriction point inactivation in combination with SHH mitogenic signaling would lead to unabated cell cycling and tumor development. Overall, in the absence of restriction point and cell cycle checkpoint safeguards, the effects of DNA damage are exacerbated as the cell cannot appropriately respond to damage.

More direct evidence for the importance of intact DDR for MB suppression follows from p53 and DDR protein ablation studies. Mice deficient for KU80 or DNA Ligase IV, proteins required for NHEJ mediated DSB repair, in a *TP53* null background, accelerates MB formation leading to shorter overall survival (140, 141). Tumorigenesis is also achieved through combined loss of p53 and other DDR pathways, including HR and BER. The absence of XRCC2, which forms a complex with Rad51 paralogs to prepare DNA filaments for HR repair, results in embryonic lethality that is rescued by *TP53* ablation, driving MB formation (142, 143). Furthermore, requirements for BER and SSB repair, pathways crucial for cisplatin adduct resolution, is made evident again by the simultaneous ablation of *TP53* and DNA Polymerase β or XRCC1 (144). *POLB* ^-/-^;*TP53* ^-/-^ and *XRCC1* ^-/-;^ *TP53* ^-/-^ mice overexpress MYCN and CDK6 and develop aggressive classical MBs resembling the SHHα subtype. The absence of genomic guardians in CGNPs amplifies their tumorigenic potential and further delineates a requirement for intact DNA damage signaling in the developing cerebellum. p53 loss is required for MB development when DNA repair mechanisms are no longer present. Where DNA damaging reagents are utilized for pediatric MB patients with defective p53, DDR signaling pathways cannot activate p53 mediated apoptosis or senescence, creating potential opportunities for therapeutic resistance.

## Targeting the p53 Response

While the p53 inactivating mutations are the most apparent method for p53 pathway dysregulation, alterations to p53 regulators or signaling effectors are also described in MB and can be therapeutically harnessed for p53 signaling modulation when combined with DNA damaging reagents. MDM2, or Double Minute 2, is an E3 ubiquitin ligase responsible for the degradation of p53 in the absence of cell stress; thus, MDM2 dysregulation may lead to inappropriate p53 degradation, resulting in cell cycle checkpoint abrogation or apoptotic resistance. And while MDM2 is only amplified in a small percentage of adult MB, manipulation of p53 levels through MDM2 inhibition could be viable in patients without MDM2 overexpression and enhance the effects of DNA damage therapies (131, 145). MDM2 ablation from the *PTCH^+/-^* model results in p53 accumulation, decreased CGNP expansion, and aberrant cerebellar foliation (146). It follows from this reasoning that disrupting the interaction between MDM2 and p53 could enhance p53 mediated apoptosis and cell cycle inhibition, made possible by MDM2 inhibitors such as nutlin (147). MDM2 inhibition by nutlin in *TP53* wild type MB cells, HDMB03, ON2-76, D341, and D283, results in the accumulation of p53, p21, and sub G1 population size; however, *TP53* mutant cells, Daoy and UW228, are unaffected (148). Additionally, when combined with a DNA intercalating agent, doxorubicin, nutlin further enhances the apoptotic and cell cycle inhibitory activity of p53 (149).

The interaction between MDM2 and p53 can also be modulated by other proteins, including BAI1, I2PP2A, and WIP1. BAI1, or Brain-specific angiogenesis inhibitor 1, inhibits angiogenesis and upregulates p53 activity through MDM2 inhibition (150, 151). Therein, *ADGRB1* (BAI1) ablation in the PTCH^+/-^ model results in substantial decreases in p53 and p21, and an approximately 50% increase in MB tumor formation. Furthermore, epigenetic reactivation of BAI1 through KCC-07, a MBD2 (Methyl-CpG-binding domain protein 2) inhibitor, potentiates the activity of p53 and cell cycle inhibition, bestowing a survival advantage in mice harboring D556 xenograft tumors (151). BAI1 epigenetic reactivation can also be achieved by inhibiting EZH2, an epigenetic transcriptional repressor responsible for H3K27 methylation (152). Application of the EZH2 inhibitor, tazemetostat, yields a mild survival advantage in *TP53* wild type MB xenograft models. Another MDM2 antagonist, I2PP2A or Phosphatase 2A Inhibitor 2 also known as SET, inhibits PP2A which dephosphorylates MDM2, rendering it incapable of degrading p53. The application of COG112 in primary SmoA1 SHH MB mouse cells inhibits I2PP2A, allowing PP2A to dephosphorylate MDM2, resulting in accumulation of p53, p21, and a decrease in cell viability of *TP53* wild type ONS-76 cells (153). Synergism between radiation or chemotherapy and I2PP2A or EZH2 inhibition remains to be determined.

A final protein of interest, WIP1/PPMD1 or wild-type p53-induced phosphatase 2, is a serine/threonine phosphatase belonging to the PP2C family, which can positively regulate p53 degradation (154, 155). WIP1 is capable of dephosphorylating p53 at Ser15 (facilitating MDM2 binding), direct upregulation of MDM2 activity, and contributing to DDR termination through pATM and γH2AX dephosphorylation. Thus, targeting WIP could amplify the ATM-Chk2 signaling axis, augmenting DNA damage recognition and p53 activation, and potentially sensitizing to DNA damaging reagents. WIP1 is overexpressed in a considerable population of MB cell lines and patient tumors as a result of chromosome 17q copy number gains, present in ~46% of patients sampled (156). And while the overexpression of WIP1 mildly abrogates accumulation of p53Ser15 phosphorylation following UV exposure and increases the expression of p21 in MB, and knockdown sensitizes to ionizing radiation in Diffuse Intrinsic Pontine Glioma (*DIPG*), the level of sensitization achievable in MB is not clear (157). Secondarily, similar to the pleiotropic nature of CK2, WIP1 is known to influence not only DDR, but also proliferation and invasiveness. Utilizing the RCAS nestin-tva mouse model, when co-infected with transgenes overexpressing SHH ligand and WIP1, 34% of mice develop spontaneous tumors compared to 8% of mice with SHH overexpression (158). Importantly, WIP1 overexpression alone was not sufficient to induce spontaneous tumor formation. WIP1 overexpression also drives expression of SHH signaling targets, Gli1 and Ptch1, and enhances proliferation in CGNPs independent of p53 inhibition (159). Finally, in both smoothened-overexpressing and *PTCH^+/-^* MB models, *PPM1D* knockout dramatically suppressed *de novo* tumor formation. Thus, targeting WIP1 could not only reduce proliferative and tumorigenic phenotypes, but also sensitize to MB DNA damaging therapies.

Historically, MB patients harboring *TP53* mutations have had a substantially worse prognosis than their WT counterparts likely due to ineffective apoptotic signaling following standard of care therapies. And while the vast majority of cancer therapies focus on inhibiting genes mediating resistance, the activation of tumor suppressors is beginning to emerge as a potential alternative. Inhibition of MDM2 oncogenes as a way to reactivate p53 is being tested in clinical trials for hematologic and solid tumors.^5ct^ Results for preclinical research for p53 activation through MDM2 and WIP1 inhibition is mixed, however, and the efficacy of these inhibitors in patients is to be determined. Secondarily, for patients with *TP53* mutations, MDM2 and WIP1 inhibition will likely have no effect because mutant p53 is dominant negative over any endogenous WT p53. Over the last two decades researchers have turned to gene therapy as a way to reintroduce p53 through recombinant viruses, a more relevant approach for *TP53* mutant tumors.^6,7ct^ This localized method to protein expression may not carry the same systemic issues as administering an MDM2 or WIP1 inhibitor, however, gene therapy is challenging and carries potentially dangerous side effects for constructs capable of mutating into self-replicating viruses. Presumably, given the role of a p53 virus would be to transiently express p53, this rids the potential for inappropriate genomic integration and cellular transformation. Thus, given the rapidly developing landscape of gene therapy, WT p53 expression could develop into a viable therapeutic option.

## Conclusions

Given the lack of success with molecular therapy in medulloblastoma and the only recently emerging impact of molecular subgrouping on patient treatment, DNA damaging therapeutics will remain the backbone therapy in MB patients for the foreseeable future. DNA damage proteins are highly conserved in their response to genotoxic therapies across patients; however, the responsiveness may be contingent upon numerous factors, including underlying genomic instability driven by MYC amplification, or the presence of secondary mutations or tumor suppressor inhibition such as *TP53* loss or mutation. These factors may drive apoptotic resistance and potentially advantageous mutagenesis. Alternatively, variations to proteins playing either a direct or indirect role in the DDR, such as increased Chk1 expression, elevated APE1 activity, or phosphorylation of AKT, can prime tumors for responsiveness to DNA damage. Together, these alterations may lead to the clonal evolution of therapeutically resistant cells from a once minority cell population such as those of the perivascular niche, ultimately resulting in patient relapse. The targeting of DDR proteins is broadly applicable even outside of a synthetically lethal context, such as with PARP dependence in *BRCA1/2* mutation positive cancers, and numerous inhibitors against PARP1, ATR, Chk1, and MDM2 are being tested in combination with standard of care radiation and chemotherapy in the setting of medulloblastoma and beyond (160–162).

## Clinical Trials Bibliography

1ct. National Library of Medicine (US). (2019, July -). Evaluation of LY2606368 Therapy in Combination With Cyclophosphamide or Gemcitabine for Children and Adolescents With Refractory or Recurrent Group 3/Group 4 or SHH Medulloblastoma Brain Tumors. ClinicalTrials.gov Identifier: NCT04023669. https://clinicaltrials.gov/ct2/show/NCT04023669

2ct. National Library of Medicine (US). (2017, July -). Olaparib in Treating Patients With Relapsed or Refractory Advanced Solid Tumors, Non-Hodgkin Lymphoma, or Histiocytic Disorders With Defects in DNA Damage Repair Genes (A Pediatric MATCH Treatment Trial). ClinicalTrials.gov Identifier: NCT03233204. https://clinicaltrials.gov/ct2/show/NCT03233204

3ct. National Library of Medicine (US). (2019, April -). Testing the Safety and Tolerability of CX-4945 in Patients With Recurrent Medulloblastoma Who May or May Not Have Surgery. ClinicalTrials.gov Identifier: NCT03904862. https://www.clinicaltrials.gov/ct2/show/NCT03904862

4ct. National Library of Medicine (US). (2017, July -). Samotolisib in Treating Patients With Relapsed or Refractory Advanced Solid Tumors, Non-Hodgkin Lymphoma, or Histiocytic Disorders With TSC or PI3K/MTOR Mutations (A Pediatric MATCH Treatment Trial). ClinicalTrials.gov Identifier: NCT03213678. https://clinicaltrials.gov/ct2/show/NCT03213678

5ct. National Library of Medicine (US). (2018, August -). Phase 1 Study of the Dual MDM2/MDMX Inhibitor ALRN-6924 in Pediatric Cancer. ClinicalTrials.gov Identifier: NCT03654716. https://clinicaltrials.gov/ct2/show/NCT03654716

6ct. National Library of Medicine (US). (2004, August - 2009, February). Gene Therapy in Treating Patients With Recurrent or Progressive Brain Tumors. ClinicalTrials.gov Identifier: NCT00004080. https://clinicaltrials.gov/ct2/show/NCT00004080

7ct National Library of Medicine (US). (2004, July - 2018, June). Gene Therapy in Treating Patients With Recurrent Malignant Gliomas. ClinicalTrials.gov Identifier: NCT00004041. https://clinicaltrials.gov/ct2/show/NCT00004041

## Author Contributions

LM and AK developed the concept of the review. SS provided clinical contextual information. DH provided radiation response in medulloblastoma expertise and advised on figure preparation. KP and JS provided Akt and DNA damage response expertise. All authors contributed to writing and approval of the final version.

## Funding

JS is supported by NCI 4R00CA204601. AK is supported by NINDS R01NS110386 and Alex’s Lemonade Stand Foundation for Childhood Cancer Research. Dr.s JS and AK are members of the Winship Cancer Institute (P30 Center Grant CA138292).

## Conflict of Interest

The authors declare that the research was conducted in the absence of any commercial or financial relationships that could be construed as a potential conflict of interest.

## Publisher’s Note

All claims expressed in this article are solely those of the authors and do not necessarily represent those of their affiliated organizations, or those of the publisher, the editors and the reviewers. Any product that may be evaluated in this article, or claim that may be made by its manufacturer, is not guaranteed or endorsed by the publisher.

## Abbreviations

WNT, Wingless; SHH, Sonic Hedgehog; DDR, DNA Damage Response; MB, Medulloblastoma; DSB, Double Strand Break; SSB, Single Strand Break; NHEJ, Non-Homologous End Joining; HR, Homologous Repair; BER, Base Excision Repair; NER, Nucleotide Excision Repair; ATM, ataxia telangiectasia mutated; ATR, ataxia telangiectasia and Rad3-related protein; Chk1, Checkpoint Kinase 1; Chk2, Checkpoint Kinase 2; PTCH, Patched; SMO, Smoothened; PARP, Poly ADP Ribose Polymerase 1; BBB, Blood Brain Barrier; CGNP, Cerebellar Granular Neural Precursor; PVN, Perivascular Niche; APE1, Apurinic/apyrimidinic endonuclease 1; CK2, Casein Kinase 2; MGMT, O6-MethylGuanine-DNA Methyltransferase; KD, Knockdown; KO, Knockout; OE, Overexpression; MRI, Magnetic Resonance Imaging; STR, Subtotal Resection; GTR, Gross Total Resection; LCA, Large Cell Anaplastic; DNV, Desmoplastic/Nodular; MBEN, medulloblastoma with extensive nodularity; LP, Lumbar Puncture; CSI, Craniospinal Irradiation; IR, Ionizing Radiation; P(n), Post-natal day (n); TBB, 4,5,6,7-Tetrabromobenzotriazole; NSCLC, non-small cell lung cancer; RTK, Receptor Tyrosine Kinase; PI3K, Phosphoinositide 3-kinase; EMT, Epithelial to Mesenchymal Transition; (M/R/N), MRE11/RAD50/NBS1.
